# Fluoxetine induces alkalinization of astroglial cytosol through stimulation of sodium-hydrogen exchanger 1: dissection of intracellular signaling pathways

**DOI:** 10.3389/fncel.2015.00061

**Published:** 2015-03-03

**Authors:** Jienan Ren, Dan Song, Qiufang Bai, Alexei Verkhratsky, Liang Peng

**Affiliations:** ^1^Laboratory of Brain Metabolic Diseases, Institute of Metabolic Disease Research and Drug Development, China Medical UniversityShenyang, China; ^2^Faculty of Life Science, The University of ManchesterManchester, UK; ^3^Achucarro Center for Neuroscience, IKERBASQUE, Basque Foundation for ScienceBilbao, Spain; ^4^University of Nizhny NovgorodNizhny Novgorod, Russia

**Keywords:** astrocytes, fluoxetine, pH_i_, NHE1, ERK_1/2_, AKT

## Abstract

Clinical evidence suggest astrocytic abnormality in major depression (MD) while treatment with anti-psychotic drugs affects astroglial functions. Astroglial cells are involved in pH homeostasis of the brain by transporting protons (through sodium-proton transporter 1, NHE1, glutamate transporters EAAT1/2 and proton-lactate co-transporter MCT1) and bicarbonate (through the sodium-bicarbonate co-transporter NBC or the chloride-bicarbonate exchanger AE). Here we show that chronic treatment with fluoxetine increases astroglial pH*_i_* by stimulating NHE1-mediated proton extrusion. At a clinically relevant concentration of 1 μM, fluoxetine significantly increased astroglial pH*_i_* from 7.05 to 7.34 after 3 weeks and from 7.18 to 7.58 after 4 weeks of drug treatment. Stimulation of NHE1 is a result of transporter phosphorylation mediated by several intracellular signaling cascades that include MAPK/ERK_1/2_, PI3K/AKT and ribosomal S6 kinase (RSK). Fluoxetine stimulated phosphorylation of ERK_1/2_, AKT and RSK in a concentration dependent manner. Positive crosstalk exists between two signal pathways, MAPK/ERK_1/2_ and PI3K/AKT activated by fluoxetine since ERK_1/2_ phosphrylation could be abolished by inhibitors of PI3K, LY294002 and AKT, triciribine, and AKT phosphorylation by inhibitor of MAPK, U0126. As a result, RSK phosphorylation was not only inhibited by U0126 but also by inhibitor of LY294002. The NHE1 phoshorylation resulted in stimulation of NHE1 activity as revealed by the NH_4_Cl-prepulse technique; the increase of NHE1 activity was dependent on fluoxetine concentration, and could be inhibited by both U0126 and LY294002. Our findings suggest that regulation of astrocytic pH*_i_* and brain pH may be one of the mechanisms underlying fluoxetine action.

## Introduction

The most primary and fundamental astroglial function lies in providing the homeostasis of the central nervous system (CNS). Numerous molecular cascades expressed (often specifically) in astrocytes control interstitial concentration of principal ions, regulate movement and metabolism of major neurotransmitters, supply neurones with energy substrates and contribute to tissue defence through, for example, secreting scavengers of reactive oxygen species (Deitmer and Rose, 2010; Rose et al., 2013; Verkhratsky et al., 2015). Astroglial cells are involved in regulation of interstitial pH by transporting both protons (mostly through sodium-proton transporter 1, NHE1, glutamate transporters EAAT1/2 and proton-lactate co-transporter MCT1) and bicarbonate (through the sodium-bicarbonate co-transporter NBC or the chloride-bicarbonate exchanger AE; see (Deitmer and Chesler, 2009; Deitmer and Rose, 2010)). The NHE1 or a solute carrier family 9 member 1, SLC9A1 represents the major pathway for H^+^ extrusion from astrocytes, in exchange for Na^+^; the NHE1 is electro-neutral antiporter with a stoichiometry 1Na^+^: 1H^+^ (Boedtkjer et al., 2012). Similarly to many other astroglial ion transporters NHE1 is regulated by a transmembrane Na^+^ gradient being thus under control of astroglial Na^+^ signaling (Kirischuk et al., 2012; Rose and Karus, 2013).

Astroglial dysfunction, pathological remodeling or reactivity contribute to pathogenesis of virtually all neurological diseases (Verkhratsky et al., 2013, 2014a) including neuropsychiatric disorders (Verkhratsky et al., 2014b). In particular evidence is constantly accumulating indicative of significant contribution of astroglia to major depression (MD). The number of glial fibrillary acidic protein (GFAP)-positive astroglial cells is conspicuously decreased in the brains of depressed patients (Rajkowska and Miguel-Hidalgo, 2007; McNally et al., 2008; Rajkowska and Stockmeier, 2013). Similarly, down-regulation of another astroglial marker, protein S100β, has been observed in the ventral prefrontal cortex of depressed suicide victims (Klempan et al., 2009). At the same time antidepressant treatment reversed these MD-associated astroglial deficits as an increase in expression of astroglial markers GFAP, S100β and aldehyde dehydrogenase 1 family, member L1 (ALDH1L1) were revealed in the post-mortem analysis (Barley et al., 2009).

Deregulation of CNS pH homeostasis is a notable feature of mood disorders. In patients with bipolar disorders the intracellular pH (pH*_i_*) in neural cells is decreased in the euthymic state as measured by ^31^P-MRS (Kato et al., 1994; Iwanaga et al., 1996; Hamakawa et al., 2004). At the same time in MD patients treated with antidepessants the pH*_i_* was not significantly different from the control group (Kato et al., 1998). Since astrocytes significantly contribute to regulation of both pH*_i_* and pH of the interstitium we performed in depth analysis of the expression and function of astroglial NHE1 by treatment with most common antidepressant drug fluoxetine. We further studies intracellular pathways involved in fluoxetine-dependent regulation of NHE1.

## Materials and methods

### Astroglial cultures

Primary cultures of astrocytes were prepared from the neopallia of the cerebral hemispheres of newborn CD-1 mice (all areas above and lateral to the lateral ventricles) by vortexing and filtering through nylon meshes with pore sizes of 80 and subsequently 10 μm as previously described (Hertz et al., 1998; Hertz, 2012), with minor modifications (Li et al., 2009). The cells were planted in 60-mm Falcon Primaria dishes or on coverslips coated with polylysine. The culture medium was a Dulbecco’s Medium Essential Medium (DMEM) with 7.5 mM glucose, initially containing 20% horse serum, and the cultures were incubated at 37°C in a humidified atmosphere of CO_2_/air (5:95%). The medium was exchanged with fresh medium of similar composition on day 3, and subsequently, every 3–4 days. From day 3, the serum concentration was reduced to 10%, and after 2 weeks, 0.25 mM dibutyryl cyclic AMP (dBcAMP) was included in the medium, which leads to a morphological and functional differentiation of astrocytes (Meier et al., 1991; Hertz et al., 1998). Characteristics, usage and advantages of these cultures has recently been authoritatively reviewed (Lange et al., 2012). Ontogenetic similarity with freshly isolated astrocytes in gene development has been reported by ourselves (Li et al., 2012b); similarly there is evidence for identical drug-induced changes in gene expression between *in vitro* and *in vivo* assessed astrocytes (Li et al., 2012a; Song et al., 2012).

### Western blotting

The protein content was determined in the homogenates by the Lowry method (Lowry et al., 1951), using bovine serum albumin (BSA) as the standard. Samples containing 75 μg protein were applied on slab gels of 10% polyacrylamide. After transfer to nitrocellulose membranes, the samples were blocked by 5% skimmed milk powder in TBS-T for 1 h, and the polyvinylidene difluoride (PVDF) membranes were incubated with the first antibody, specific to specific to p-ERK, ERK, p-ribosomal S6 kinase (RSK) or β-actin for 2 h at room temperature, or specific to p-AKT or AKT overnight at 4°C. After washing, the blots were incubated with peroxidase-conjugated affinity-purified goat-anti-mouse or goat-anti-rabbit horseradish antibody for 2 h. Staining was visualized by ECL detection reagents. Digital images obtained using Gel-Imaging System (Tanon 4200, Shanghai, China). Optical density for each band was quantified using the Window AlphaEase TM FC 32-bit software.

### Immunoprecipitation and Western blotting for NHE1

The phosphorylation level of NHE1 was determined by immunoprecipitation with an antibody recognizing the phospho-Ser 14-3-3 which binds to p-NHE1 as previously reported (Snabaitis et al., 2006; Luo et al., 2007). After homogenization, whole cell lysates (1,000 μg) were incubated with 4 μl of anti-mouse phospho-Ser 14-3-3 protein binding motif antibody for 12 h at 4°C. Thereafter 50 μl of washed Protein A/G PLUS—agarose bead slurry was added, and the mixture was incubated for another 2 h at 4°C. The agarose beads were collected by pulsing centrifuge (5 s in the microcentrifuge at 14,000 g), the supernatant drained off and the beads boiled for 5 min. Thereafter, the supernatant was collected by pulsing centrifuge and the entire immunoprecipitates were subjected to 8% SDS-polyacrylamide gel electrophoresis (PAGE). After transfer to PVDF memebranes, the membranes were incubated with the antibody specific to NHE1 at a 1:500 dilution overnight at 4°C and anti-mouse antibody at a 1:2000 dilution for 2 h at room temperature.

### Monitoring of pH*_i_*

The fluorescent pH-sensitive indicator BCECF-AM and an Olympus IX71 live cell imaging fluorescence microscope (Tokyo, Japan) were used to detect pH*_i_* in individual cells as described by Grant and Acosta (1997) and Manning and Sontheimer (1999). After washing with 4-(2-hydroxyethyl)-1-piperazineethanesulfonic acid (HEPES) buffer (NaCl: 140 mM; KCl: 5.36 mM; MgSO_4_: 0.81 mM; CaCl_2_: 1.27 mM; KH_2_PO_4_: 0.44 mM; Na_2_HPO_4_: 0.33 mM; glucose: 5.55 mM; HEPES: 20 mM; pH: 7.4), cells were loaded with 5 μM BCECF-AM for 30 min at 37°C. After washing twice with HEPES buffer, fluorescence was excited at 440 and 490 nm and emission monitored at 530 nm. The fluorescence ratio (490/440 nm) was determined and calibrated to yield pH*_i_* values by interpolation between the measured fluorescence ratios in a similar culture after 20 min of exposure to one of 3 calibration buffers (pH: 6.3, 6.9 or 7.5; KCl: 130 mM; MgCl_2_: 1 mM; HEPES: 15 mM; MES: 15 mM). The calibration buffer also contained 56 μM nigericin, an H^+^/K^+^ exchanger, in order to equilibrate pH*_i_* with extracellular pH. Steady-state pH*_i_* was determined as the average of five individual readings, each from six 20 s intervals (Song et al., 2008, 2013).

Acid challenge was performed by the NH_4_Cl-prepulse technique (Boron and De Weer, 1976; McAlear and Bevensee, 2004). Cells were washed twice after BCECF-AM loading. Intracellular fluorescence ratio was determined at 25 s interval. Cells were bathed in HEPES buffer during the first four cycles, exposed to 20 mM NH_4_Cl added to the HEPES buffer with an equiosmolar reduction in the NaCl concentration during the next five cycles, and again incubated in HEPES buffer during the last twenty-seven 25 s intervals. Recovery of pH*_i_* (ΔpH*_i_*) during the last twenty-seven 25 s intervals was calculated by subtracting the pH*_i_* after about 2 min (five cycles) exposure to NH_4_Cl from the pH*_i_* measured every minute during the recovery for total of 10 min, and NHE activity was determined as ΔpH*_i_*/Δt (Song et al., 2008, 2013).

### Chemicals

Chemicals for culture media and dBcAMP were purchased from Sigma (St. Louis, MO, USA) and horse serum from Invitrogen (Carlsbad, CA, USA). Fluoxetine, SB204741 (N-(1-methyl-5-indolyl)-N′-(3-methyl-5-isothiazolyl) urea), triciribine hydrate and first antibodies, raised against β-actin were also purchased from Sigma (St. Louis, MO, USA). The U0126 (1,4-diamino-2,3-dicyano-1,4-bis[2-aminophenylthio]butadiene) and LY294002 (2-(4-Morpholinyl)-8-phenyl-4H-1-benzopyran-4-one) were obtained from Calbiochem (La Jolla, CA, USA). Santa Cruz Biotechnology (Santa Cruz, CA, USA) supplied first antibodies, raised against ERK (K-23), sc-94, against phosphorylated ERK (E-4), sc-7383, against phosphorylated AKT1/2/3 (Ser 473), sc-33437 and against phosphorylated RSK1/2 (Thr 359/Ser 363), sc-12898-R. Protein A/G PLUS-agarose, sc-2003 were also purchased from Santa Cruz Biotechnology (Santa Cruz, CA, USA). The rabbit polyclonal antibody against AKT and phosphor-(Ser) 14-3-3 binding motif antibody were purchased from Cell Signaling Technology (Danvers, MA, USA). Chemicon (Temecula, CA, USA) supplied the first antibody, raised against NHE1. The second antibody goat anti-mouse IgG HRP conjugate (W4021) was from Promega (Madison, WI, USA) and goat anti-rabbit IgG HRP conjugate (sc-2004) from Santa Cruz Biotechnology (Santa Cruz, CA, USA). ECL detection reagents were from Amersham Biosciences, Buckinghamshire, UK. Invitrogen Corp. (Carlsbad, CA, USA) provided BCECF-AM, an acetoxymethyl (AM) ester of 2′,7′-bis(2-carboxyethyl)-5(6)-carboxyfluorescein (BCECF).

### Statistics

Differences between multiple groups were evaluated by one-way analysis of variance (ANOVA) followed by Fisher’s least significant difference (LSD) multiple comparison test for unequal replications. The level of significance was set at *P* < 0.05.

## Results

### Treatment with fluoxetine increases astroglial pH*_i_*

**Figure 1:**
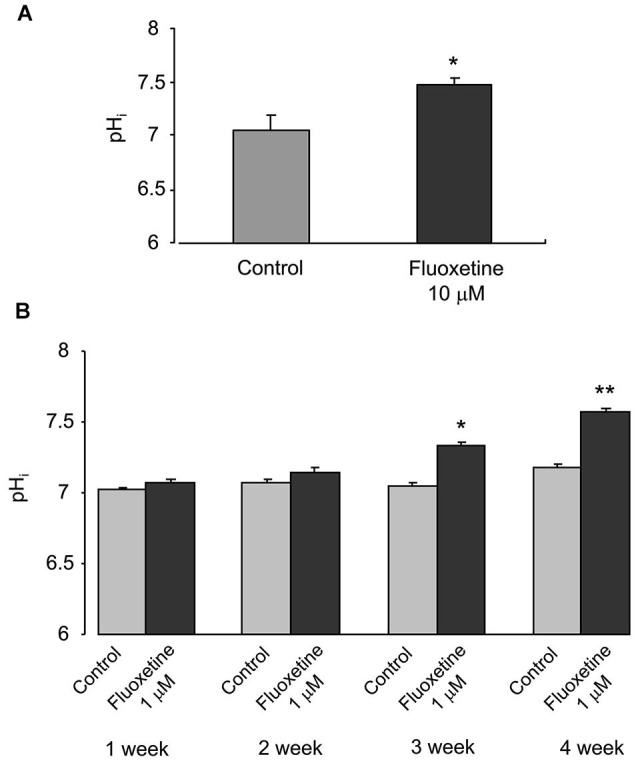
**Chronic treatment with fluoxetine increases pH*_i_* in primary cultured astrocytes. (A)** The pH*_i_* values measured in control cells or in cells treated with 10 μM fluoxetine for 3 days. All results are means of 32 cells from 3 coverslips. SEM values are indicated by vertical bars. *Indicates statistically significant (*P* < 0.05) difference from control group. **(B)** The pH*_i_* values were measured from control cells and cells treated with 1 μM fluoxetine for 1, 2, 3 or 4 weeks. All results are means of 114 to 178 cells from 6 to 9 coverslips (1 week: control, *n* = 156 cells from 8 separate coverslips; fluoxetine, *n* = 178 cells from 9 coverslips. 2 week: control, *n* = 167 cells from 9 separate coverslips; fluoxetine, *n* = 119 cells from 6 coverslips. 3 week: control, *n* = 134 cells from 7 separate coverslips; fluoxetine, *n* = 165 cells from 8 coverslips. 4 week: control, *n* = 114 cells from 6 separate coverslips; fluoxetine, *n* = 153 cells from 8 coverslips.). SEM values are indicated by vertical bars. *Indicates statistically significant (*P* < 0.05) difference from control group at the same treatment period; **Significant (*P* < 0.05) difference from all other groups.

Chronic treatment with fluoxetine caused a concentration- and time-dependent intracellular alkalinization (Figure 1). In astrocytes exposed to 10 μM fluoxetine for 3 days the pH*_i_* inreased from 7.05 ± 0.13 (*n* = 32) to 7.48 ± 0.06 (*n* = 32) after 3 days of treatment (Figure 1A). When cells were incubated with 1 μM fluoxetine (which concentration is closer to therapeutically relevant) the pH*_i_* did not change during the first week of treatment (Figure 1B), with a trend towards an increase after 2 weeks of treatment, which was not significant. After 3 and 4 weeks of treatment, pH*_i_*, however, increased significantly. Chronic treatment with 1 μM fluoxetine for 3 weeks increased pH*_i_* from 7.05 ± 0.02 (*n* = 134) to 7.34 ± 0.02 (*n* = 165), and for 4 weeks from 7.18 ± 0.02 (*n* = 114) to 7.58 ± 0.01 (*n* = 153). Alkalinization of astroglial cytoplasm indicates, most likely, an increase in H^+^ extrusion which, arguably, may result from an increase in activity of NHE1 that represents the main pathway for proton efflux.

### Fluoxetine induces phosphorylation of NHE1

**Figure 2:**
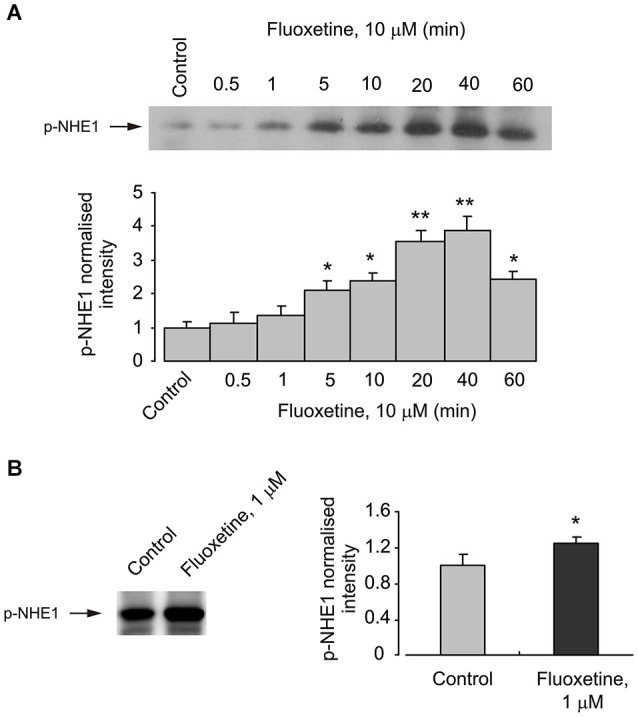
**Acute effect of fluoxetine on phosphorylation of NHE1. (A)** Cells were incubated with 10 μM fluoxetine for 0 (Control), 0.5, 1, 5, 10, 20, 40 or 60 min. *Top*: Immunoblots from representative experiments. Bands of 105 kDa represent p-NHE1. Similar results were obtained from three independent experiments. *Bottom*: Average NHE1 phosphorylation was quantified as scanned band intensity of NHE1 and normalized vs. band intensity from the control group. SEM values are indicated by vertical bars. *Indicates statistically significant (*P* < 0.05) difference from control, 0.5, 1, 20 and 40 min groups. **Indicates statistically significant (*P* < 0.05) difference from control, 0.5, 1, 5, 10, and 60 min groups. **(B)** Cells were incubated with 0 (Control) or 1 μM fluoxeine for 20 min. *Left*: Immunoblots from representative experiments. Bands of 105 kDa represent p-NHE1. Similar results were obtained from five independent experiments. *Right*: Average NHE1 phosphorylation was quantified as scanned band intensity of NHE1 and normalized vs. band intensity from the control group. SEM values are indicated by vertical bars. *Indicates statistically significant (*P* < 0.05) difference from control group.

The activity of NHE1 is known to be regulated by the transporter phosphorylation (Moor and Fliegel, 1999). This phosphorylation indeed takes place in cultured astrocytes exposed to fluoxetine, as demonstrated on Figure 2. Phosphorylation of NHE1 in cells treated with 10 μM fluoxetine occurs rapidly reaching its maximum after 40 min and beginning to decline after 60 min (Figure 2A). Exposure of cultured astrocytes to 1 μM fluoxetine also induced NHE1 phosphorylation (Figure 2B).

### Interaction between PI3K/AKT and MAPK/ERK

**Figure 3:**
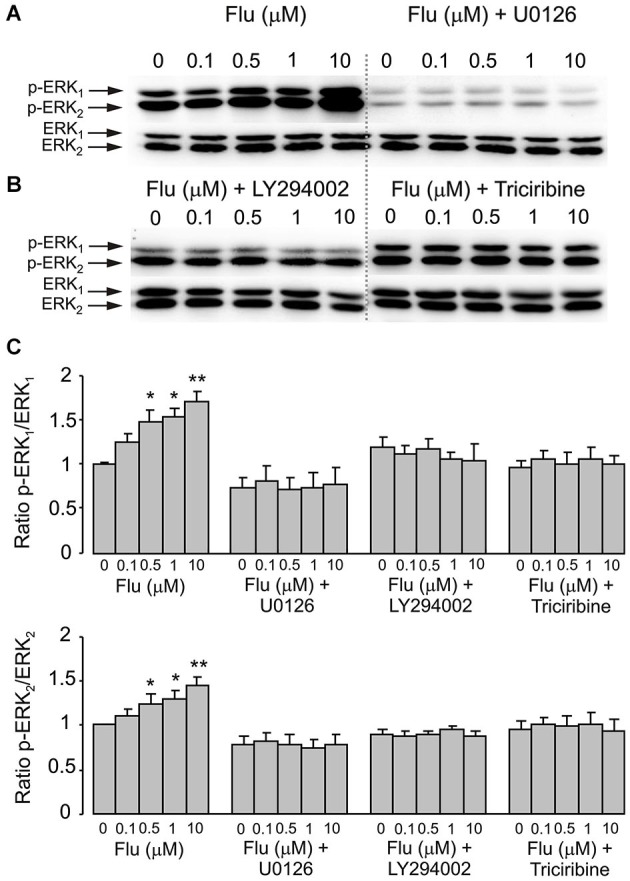
**ERK_1/2_ phosphorylation induced by fluoxetine requires MEK and AKT phosphorylation. (A,B)** Immunoblots from representative experiments. **(A)** after pre-treatment in serum-free medium with or without 10 μM of MEK inhibitor U0126, 25 μM of PI3K inhibitor LY294002 or 10 μM of AKT inhibitor triciribine **(B)** for 15 min, cells were incubated for 20 min with 0 (Control), 0.1, 0.5, 1, 10 μM fluoxetine. Bands of 44 and 42 kDa represent p-ERK_1_ (phosphorylated ERK_1_) and p-ERK_2_ (phosphorylated ERK_2_), respectively (upper rows), or total ERK_1_ and ERK_2_ (lower rows). Similar results were obtained from five independent experiments. **(C)** Average ERK phosphorylation was quantified as ratios between p-ERK_1_ and ERK_1_ (*Top*) and between p-ERK_2_ and ERK_2_ (*Bottom*). Ratios between p-ERK_1_ and ERK_1_ or p-ERK_2_ and ERK_2_ were normalized to the control. SEM values are indicated by vertical bars. *Indicates statistically significant (*P* < 0.05) difference from control group, 0.1 and 10 μM fluoxetine groups and fluoxetine plus U0126, LY294002 or triciribine groups. **Indicates statistically significant (*P* < 0.05) difference from all other groups.

**Figure 4:**
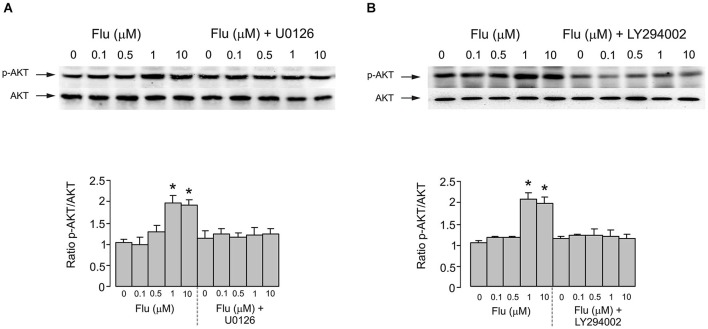
**AKT phosphorylation induced by fluoxetine requires MEK and PI3K phosphorylation. (A)** After pretreatment in serum-free medium with or without 10 μM of MEK inhibitor U0126 for 15 min, cells were incubated for 20 min with 0 (Control), 0.1, 0.5, 1, 10 μM fluoxetine. *Top*: Immunoblots from representative experiments. Bands of 60 kDa represent p-AKT (Ser473) (upper rows), or total AKT (lower rows). Similar results were obtained in five independent experiments. *Bottom*: Average AKT phosphorylation was quantified as ratios between p-AKT and AKT and normalized to controls. SEM values are indicated by vertical bars. *Indicates statistically significant (*P* < 0.05) difference from control group, 0.1 and 0.5 μM fluoxetine groups and fluoxetine plus U0126 groups. **(B)** After pretreatment in serum-free medium with or without 25 μM of PI3K inhibitor LY294002 for 15 min, cells were incubated for 20 min with 0 (Control), 0.1, 0.5, 1, 10 μM fluoxetine. *Top*: Immunoblots from representative experiments. Bands of 60 kDa represent p-AKT (Ser473) (upper rows), or total AKT (lower rows). Similar results were obtained in five independent experiments. *Bottom*: Average AKT phosphorylation was quantified as ratios between p-AKT and AKT and normalized to controls. SEM values are indicated by vertical bars. *Indicates statistically significant (*P* < 0.05) difference from control group, 0.1 and 0.5 μM fluoxetine groups and fluoxetine plus LY294002 groups.

Pharmacological experiments described in previous section highlighted the possible role for PI3K/AKT and MAPK/ERK signaling pathways. Acute (20 min) treatment of astroglial cells with fluoxetine indeed triggered ERK_1/2_ phosphorylation in concentration-dependent manner (Figure 3). The fluoxetine-dependent ERK_1/2_ phosphorylation was inhibited by 10 μM U0126 (Figure 3A). Similarly ERK_1/2_ phopshorylation was suppressed by 25 μM of LY294002, the PI3K inhibitor and by 10 μM triciribine, the AKT inhibitor (Figure 3B) indicating signaling downstream of PI3K and AKT. Fluoxetine at 1 and 10 μM increased AKT phosphorylation (Figure 4). Fluoxetine-dependent AKT phosphoryation was abolished by 25 μM LY294002 (Figure 4A) and by 10 μM U0126 (Figure 4B).

### RSK phosphorylation

**Figure 5:**
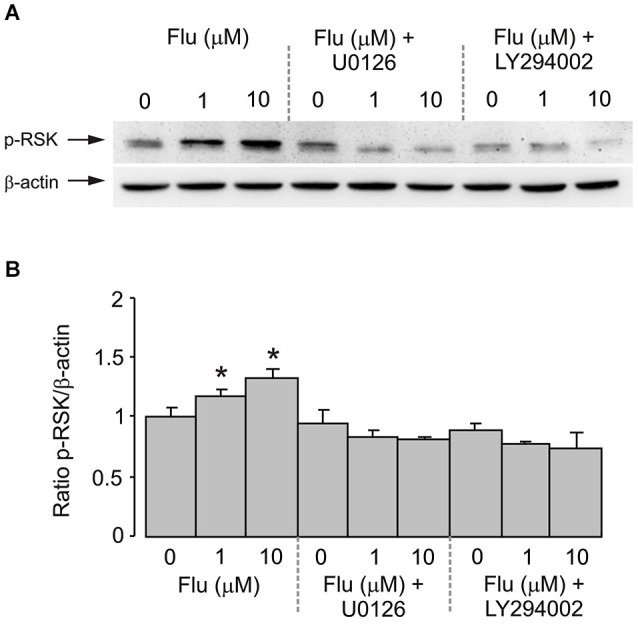
**RSK phosphorylation induced by fluoxetine requires MEK and PI3K phosphorylation**. After pretreatment in serum-free medium with or without 10 μM of MEK inhibitor U0126 or 25 μM of PI3K inhibitor LY294002 for 15 min, cells were incubated for 20 min with 0 (Control), 1, 10 μM fluoxetine. **(A)** Immunoblots from representative experiments. Bands of 90 and 46 kDa represent p-RSK (phosphorylated RSK) (upper row) and β-actin (lower row), respectively. Similar results were obtained in four independent experiments. **(B)** Average RSK phosphorylation was quantitated as ratios between p-RSK and β-actin and normalized to controls. SEM values are indicated by vertical bars. *Indicates statistically significant (*P* < 0.05) difference from all other groups.

Fluoxetine also potentiated the phosphorylation of RSK in cultured astrocytes. Treatment of cells with 1 and 10 μM fluoxetine for 20 min induced a significant increase of RSK phosphorylation (Figure 5A). The level of RSK phosphorylation was significantly higher at 10 μM than at 1 μM. The effect of fluoxetine was abolished by 10 μM of U0126 and by 25 μM of LY294002 (Figure 5B).

### NHE1 activity

**Figure 6:**
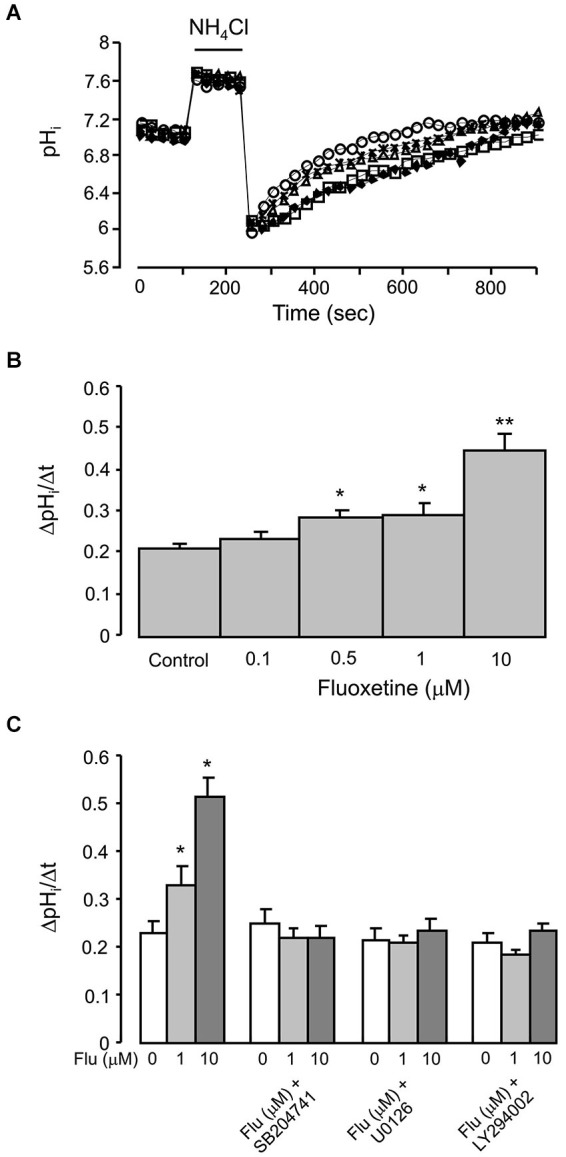
**Acute effect of fluoxetine on recovery of pH*_i_* from an acid load in primary cultured of astrocytes, which were exposed to 20 mM NH_4_Cl for 2 min. (A)** A representative experiment showing recovery of pH*_i_* after 20 min of incubation with 0 (control; diamonds), 0.1 (squares), 0.5 (triangles), 1 (asterisks) or 10 μM (circles) fluoxetine. **(B)** Recovery of pH*_i_* is presented as ΔpH*_i_*/Δt. All results are means of ΔpH*_i_*/Δt of 60 to 120 cells from 4 to 6 coverslips (control, *n* = 99 cells from 5 separate coverslips; 0.1 μM fluoxetine, *n* = 77 cells from 4 coverslips; 0.5 μM fluoxetine, *n* = 60 cells from 4 separate coverslips; 1 μM fluoxetine, *n* = 72 cells from 6 separate coverslips; 10 μM fluoxetine, *n* = 120 cells from 6 separate coverslips). SEM values are indicated by vertical bars. *Indicates statistically significant (*P* < 0.05) difference from control, 0.1 and 10 μM groups; **Significant (*P* < 0.05) difference from all other groups. **(C)** After pretreatment with or without 200 nM of 5-HT_2B_ receptor antagonist SB204741, 10 μM of MEK inhibitor U0126 or 25 μM of PI3K inhibitor LY294002 for 15 min, cells were incubated for 20 min with 0 (Control), 1, 10 μM fluoxetine. Recovery of pH*_i_* is presnetd as ΔpH*_i_*/Δt. All results are means of ΔpH*_i_*/Δt of 36 to 90 cells from 3 to 5 coverslips (control, *n* = 90 cells from 5 separate coverslips; 1 μM fluoxetine, *n* = 90 cells from 5 separate coverslips; 10 μM fluoxetine, *n* = 90 cells from 5 separate coverslips; SB204741, *n* = 58 cells from 3 separate coverslips; SB204741 plus 1 μM fluoxetine, *n* = 75 cells from 4 separate coverslips; SB204741 plus 10 μM fluoxetine, *n* = 65 cells from 3 separate coverslips; U0126, *n* = 43 cells from 3 separate coverslips; U0126 plus 1 μM fluoxetine, *n* = 48 cells from 3 separate coverslips; U0126 plus 10 μM fluoxetine, *n* = 36 cells from 3 separate coverslips; LY294002, *n* = 82 cells from 5 separate coverslips; LY294002 plus 1 μM fluoxetine, *n* = 82 cells from 5 separate coverslips; LY294002 plus 10 μM fluoxetine, *n* = 88 cells from 5 separate coverslips). SEM values are indicated by vertical bars. *Indicates statistically significant (*P* < 0.05) difference from all other groups.

Finally, we quantified NHE1 activity by measuring the rate of pH*_i_* changes in individual cells following acidification induced by 20 mM NH_4_Cl (Figure 6). In cells pretreated (for 20 min before measurements) with 0.1, 1, or 10 μM of fluoxetine the pH*_i_* recovered faster; and the rate of recovery increased with an increase in fluoxetine concentration (Figure 6A). Fluoxetine-dependent increase in the activity of NHE1 was abolished by 200 nM SB204741, an antagonist of 5-HT_2B_ receptor, by 10 μM of U0126 or by 25 μM of LY294002 (Figure 6B).

## Discussion

Proton homeostasis and maintenance of intra- and extracellular pH in the brain is critically important for normal activity of neural cells and therefore for brain functions. Decrease in brain pH is associated with variety of pathological conditions, such as ischemia and epilepsy (Katsura and Siesjo, 1998). The pH of the extracellular fluid is affected by numerous pH regulating systems localized in neurones and neuroglia (for review, see McAlear and Bevensee, 2004; Deitmer and Chesler, 2009). Astroglia play an important role in regulation of interstitial pH and pH*_i_* by their acid-loading, acid-extruding and bicarbonate transporting systems (McAlear and Bevensee, 2004; Deitmer and Chesler, 2009). Previously, we have reported that chronic treatment of astrocytes with three antibipolar drugs induced intracellular alkalization (Song et al., 2008, 2013). Specifically, lithium acutely stimulated proton efflux through NHE1 by direct action at the extracellular site (Song et al., 2008); whereas carbamazepine and valproic acid up-regulated expression of electrogenic Na^+^/HCO_3_^−^ co-transporter NBCe1/SLC4A4 (Song et al., 2013). In the present paper we performed systematic investigation of the effects of fluoxetine on pH regulation and intracellular signalling cascades in astrocytes. In particular, we investigated effects of fluoxetine on (i) the pH*_i_*; (ii) the NHE1 phosphorylation; (iii) the interaction of MAPK/ERK_1/2_ and PI3K/AKT signal pathways; (iv) the RSK phosphorylation; and (v) the NHE1 activation following acidic stress.

**Figure 7:**
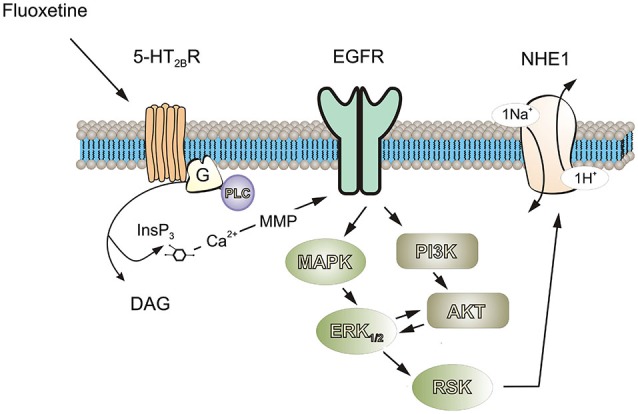
**Possible signaling cascades mediating fluoxetine-induced stimulation of ptoron transport in astrocytes**. Fluoxetine acts as an agonist at 5-HT_2B_ receptor. Stimulation of 5-HT_2B_ receptor activates phospholipase C (PLC), generating diacylglycerol (DAG) and inositol 1,4,5-trisphosphate (IP3) and leading to an increase of free cytosolic calcium concentration and stimulation of matrix metalloproteinases (MMPs), which in turn induced transactivation of EGF receptors. The activation of EGF receptor stimulates MAPK/ERK and PI3K/AKT signaling cascades with a positive feedback loop existing between these two pathways. Ninety-kDa ribosomal S6 kinase (RSK or p90*^RSK^*), downstream of ERK can increase the activity of NHE1 by phosphorylation of Ser-703.

As expected, both NHE1 and RSK were phosphorylated by acute treatment with fluoxetine, suggesting RSK was involved in NHE1 phosphorylation. This was suppressed by inhibition of either MAPK/ERK_1/2_ or PI3K/AKT signaling pathways, indicating the interaction between them is required, although RSK is positioned downstream of MAPK/ERK_1/2_ (Figure 7). The NHE1 activity measured by NH_4_Cl challenge was blocked by inhibition of either MAPK/ERK_1/2_ or PI3K/AKT pathway, and by an antagonist of 5-HT_2B_ receptor, demonstrated that the stimulation of NHE1 by fluoxetine is mediated by 5-HT_2B_ receptor. Previously, we reported that fluoxetine acts as agonists of 5-HT_2B_ receptors in astrocytes (Kong et al., 2002). All three 5-HT_2_ receptors, 5-HT_2A_, and 5-HT_2B_ and 5-HT_2C_ receptors are G_*q*/11_ protein-coupled, and stimulation of these receptors activates phospholipase C (PLC), thus generating diacyglycerol (DAG) and inositol 1,4,5-trisphosphate (InsP_3_). The fluoxetine-induced and 5-HT_2B_ receptor-mediated ERK_1/2_ phosphorylation as well as the effect of fluoxetine on epidermal growth factor receptor (EGFR) phosphorylation could be abolished by (i) AG1478, an inhibitor of the EGFR tyrosine kinase and by (ii) GM6001, a potent and broad-based inhibitor of Zn^2+^-activated metalloproteinases (Li et al., 2008), suggesting the role of EGFR transactivation. Fluoxetine also stimulates PI3K/AKT signal pathway by EGFR transactivation in astrocytes (Peng and Huang, 2012). The 90 kDa RSK is the downstream effector of ERK_1/2_. The RSK belongs to highly conserved Ser/Thr kinases that are involved in many cellular processes (Anjum and Blenis, 2008), and is also known as a NHE1 kinase (Vik and Ryder, 1997).

We found that fluoxetine at clinically relevant concentrations induced phosphorylation of both ERK_1/2_ and AKT in astrocytes. Conceptually, the PI3K/AKT and Raf/MAPK/ERK_1/2_ represent two parallel signal pathways, although, the interactions between PI3K/AKT and Raf/MAPK/ERK_1/2_ cascades may occur at several different stages and could be either positive or negative. In the present work we found that PI3K/AKT and MAPK/ERK_1/2_ signaling potentiated each other (Figure 7). Previously, we reported that either PI3K or AKT may stimulate the activity of Raf, MAPK or ERK_1/2_ in ammonium-induced ERK_1/2_ phosphorylation in astrocytes, since ERK_1/2_ phosphorylation was inhibited by both the MAK inhibitor U0126 and PI3K/AKT inhibitor, LY294002 (Dai et al., 2013). A similar pattern has been observed for effects (i) of insulin or insulin-like growth factor-1 (IGF-1) on skeleton muscle cells (Cross et al., 1994) or insulin receptor-transfected Chinese hamster ovary cells (Welsh et al., 1994); (ii) of platelet-activating factor on guinea pig neutrophils (Ferby et al., 1994); (iii) of interleukin-2 on T-lymphocytes (Karnitz et al., 1995); and (iv) of platelet-derived growth factor on fibroblasts and T-lymphocytes (Grammer and Blenis, 1997). Although PI3K is one of the major downstream signals of RAS small GTPases (Castellano and Downward, 2011), there is also evidence that PI3K activates MAPK/ERK_1/2_ pathway at/or upstream of RAS (Hawes et al., 1996). To the best of our knowledge, our finding is the first report that MAPK/ERK_1/2_ potentiates the PI3K/AKT signaling pathway.

The pH*_i_* increases in astrocytes chronically treated with fluoxetine in a time- and concentration-dependent manner. This reflects changes in pH homeostasis that may influence brain pH and astrocytic functions and may contribute to the drug action. Changes in pH*_i_* significantly affects astroglial physiology. Addition of lactate acid or a decrease of pH*_i_* inhibits communications through gap junction and facilitates internalization of Cx43 in astrocytes (Anders, 1988; Duffy et al., 2004). Astrocytic proliferation is also regulated by changes in pH*_i_* (Pappas et al., 1994). A decrease in pH*_i_*, for instance, inhibited conversion of inactive phosphorylase b to active phosphorylase a, thus decreasing glycogenolysis (Danforth, 1965). In the brain, glycogen is located almost exclusively in astrocytes (Ibrahim, 1975); glycogen is rapidly consumed along with enhanced neuronal activity (for example during whisker stimulation) (Swanson et al., 1992), this process contributing to neuronal energetics. The glycogenolysis is similarly important for many signaling processes in astrocytes (Hertz et al., 2015). It is ideal for such a role because it does not require phosphorylation (Hertz et al., 2007); and it is regulated by physiological signals including [Ca^2+^]*_i_* rises (Ozawa, 1972) by various transmitters and by increases in extracellular K^+^ concentration (Hof et al., 1988; Magistretti, 1988; Obel et al., 2012). Astroglial release of ATP release stimulated by high K^+^, glutamate and adenosine is also dependent on glycogenolysis (Xu et al., 2014). In summary, fluoxetine-induced intracellular alkalization may benefit astrocytic gap junction and signal transduction. It will be interesting to know whether these astrocytic functions are deregulated in depressed animals.

## Author and contributors

LP conceived and designed experiments; JR, DS, QB collected and analyzed the data; LP and AV interpreted the data and wrote the paper. All authors commented on the manuscript and have approved the final version.

## Conflict of interest statement

The authors declare that the research was conducted in the absence of any commercial or financial relationships that could be construed as a potential conflict of interest.
